# Roots to Grow and Wings to Fly: An Ethnography of Psychosocial Development in Adolescent Performance Sport

**DOI:** 10.3390/sports10040048

**Published:** 2022-03-22

**Authors:** Sergio Lara-Bercial, Jim McKenna

**Affiliations:** 1 Research Centre for Sport Coaching, Carnegie School of Sport, Leeds Beckett University, Leeds LS6 3QT, UK; j.mckenna@leedsbeckett.ac.uk; 2 Global Coaching Office, International Council for Coaching Excellence, Leeds Beckett University, Leeds LS6 3QT, UK

**Keywords:** positive youth development, youth sport, realist evaluation, life skills, personal development

## Abstract

This study aimed to explore the potential for sport to support psychosocial development in young people in a youth performance setting using a novel realistic evaluation approach. Part 1 of this two-paper series published in this Special Issue identified the programme theories—how the programme is supposed to work. A wide and deep network of context, generative mechanisms and outcomes responsible for psychosocial development in this youth performance basketball club emerged. The first paper also concluded that the outcomes and the experience are highly contextual and individualised. In this second part, the stakeholder’s programme theories were tested during a full-season ethnography of the same club. Immersion in the day-to-day environment generated a fine-grain analysis of the processes involved, including: (i) sustained attentional focus; (ii) structured and unstructured skill-building activities; (iii) deliberate and incidental support; and (iv) feelings indicating personal growth. Personal development in and through sport is thus shown to be conditional, multi-faceted, time-sensitive and idiosyncratic. The findings of this two-part study are considered to propose a model of psychosocial development in and through sport. This heuristic tool is presented to support sport psychologists, coaches, club administrators and parents to deliberately create and optimise developmental environments.

## 1. Introduction

Sport has the potential to support positive outcomes in young people. Part 1 of this two-paper series provided a detailed review of existing literature in this area and of its shortcomings. By way of a summary, extant search has positively linked sport participation to physical and mental health and wellbeing. Likewise, prior studies have also highlighted the capacity for sport to foster psychosocial development in young people. However, our review also showed that previous studies have tended to evaluate purpose-built interventions, leaving regular organised sport relatively overlooked. Moreover, previous work has tended to concentrate on a narrow range of outcomes and methodologies. To address these gaps, we conducted a season-long ethnography of a youth performance sport club using a novel realist evaluation (RE) approach [1,2]. This methodology has never been used before in the study of psychosocial development in youth sport.

RE typically aims to establish how well a given purpose-built social intervention achieves its expected outcomes. RE’s unique features have particular utility in the study of psychosocial development in organized youth sport when sport is construed and positioned as an organic social intervention embedded within a complex social system. In other words, these are environments where, with or without the awareness, intention and volition of those within it, a series of mechanisms interact, leading to explicit, implicit, desired and undesired developmental outcomes for all involved. RE emphasises the fundamental role of theory in driving social research and the importance of looking beyond quantification and correlation to shed light on the generative mechanisms affecting choices, behaviours and, ultimately, the outcomes of a given intervention. RE is thus concerned with the notion and nature of causality.

Part 1 of this two-paper series focused on identifying key stakeholder programme theories (PTs) related to psychosocial development in a youth performance basketball club in the North of England. Former and current players (*n* = 16), their parents (*n* = 18), and current coaches (*n* = 5) took part in in-depth semi-structured interviews and focus groups. Current players and parents belonged to the Under 13 (U13) and Under 16 (U16) squads. The analysis elicited a deep network of interrelated positive and negative developmental outcomes, generative mechanisms and salient features of the context. The findings also highlighted the complex and systemic nature of the PYD process, where factors and elements combine, interact and catalyse in multiple, non-linear ways to produce highly individualised outcomes.

In line with the adopted RE approach, this second part of the study tested the previously proposed stakeholder PTs in the day-to-day workings of the club. This ‘coalface’ analysis was deployed to establish, or refute, the validity of the respective PTs and reveal previously unseen components and relationships that may challenge the espoused accounts. Layder [3] describes this “adaptive theory” as the never-ending cycle of refinement of existing theory based on new emerging data to arrive at new insights [4]. This second phase also aimed to develop and present an integrative model of psychosocial development to support sport psychologists, coaches, club administrators and parents to create and optimise other developmental environments.

## 2. Study Design and Methods

### 2.1. Study Design

As part of the case study methodology described in part 1, the second part of the study was comprised of the lead author conducting a full-season ethnography of a youth performance basketball club. To the best of our knowledge, no ethnography accounts of psychosocial development in youth sport exist in the literature. Wolcott [5] describes ethnography as the process of learning about culture as manifested through distinct and observable patterns of socially shared behaviours. Ethnographers, therefore, aim to gain a deep understanding of the functioning of social groups and of the implications for the group and its members. However, this understanding of culture must come from the viewpoint of the group members [6]. Therefore, central to all different interpretations of ethnography is that it includes “a commitment to first-hand experience” [7] (p. 34) in the shape of “direct and sustained social contact with agents” [8] (p. 5).

The study used Wolcott’s experiencing-enquiring-examining framework [5]. Experiencing revolves around participant observation and, in some cases, the researchers’ own participation. Enquiring requires that we ask participants about “what’s going on”(p. 49). Examining involves the study of documents and artefacts produced in the environment. As a result, ethnographic researchers need to remain flexible in using a wide variety of research methods depending on how social life unfolds [9].

Notwithstanding this need for methodological elasticity, Wolcott’s framework indicates that ethnographers must start with intent: the researcher creates in advance a clear understanding of what is to be studied. For Wolcott, “fieldwork must be preceded by mindwork” (p. 70). From this perspective, ethnography acts more as a philosophy of how to conduct research rather than a method or technique to be employed [9]. Ethnography is thus theory-driven yet dynamic and adaptable to deal with the varying demands of the ‘live’ environment. An ethnographic approach thus fits well within the broader RE theoretical framework of the study and its focus on explanation and causality in the complex and multi-layered context of a social intervention.

### 2.2. Study Setting

As described in part 1, a youth performance development basketball club in North West England was identified following a criterion-based sampling process. Criteria included an environment featuring: (i) high intensity and frequency of participant engagement combined with high future stakes (i.e., a professional career); (ii) a hybrid club ethos combining community- and performance-based values and provision, leading to a wide range of experiences and interactions; and (iii) an explicit humanistic philosophy combined with a prolonged high level of success at the national and international level. It is important to note the lead researcher’s familiarity with the setting, where he has coached for the last 20 years. The pros and cons of this element were carefully weighed regarding the balance of insider versus outsider positionality; the research team decided that the benefits outweighed the potential negative effects. Note also that none of the research subjects were or had ever been coaches coached by the lead author. On these bases, permission to progress with the study was provided by the club Chairman. Approval was subsequently granted by the Ethics Committee of Leeds Beckett University.

### 2.3. Participants

The study focused on the U13 and U16 boys squads. A single-gender approach was chosen due to the female section of the club being nearly completely run separately from the male side. The squads were comprised of 15 players each, with fluctuations over the course of the season due to injuries and late additions. Due to the location of the club in an inner-city and its performance-based nature, the club attracts a broad range of young people and families from varying socioeconomic statuses and ethnicities. Whereas part 1 focused on a small number of participants from each squad, this second part took into consideration the experiences of all squad members and their families and coaches (for full details of participants, see Paper 1 of the series and its Supplementary Materials from Paper 1). Moreover, given the full immersion of the lead author within the club, other inhabitants of the setting also contributed to the study by interacting with the researcher purposefully and explicitly, or in passing, during his visits to the club.

### 2.4. Data Collection

The lead author spent a full season (August to May) in the field. The emerging collection of context-mechanism-outcome networks (CMONs) described in part 1 was used to guide the researcher during the immersive period. The researcher purposefully sought to ascertain, refute and/or refine the presence of these networks in the club environment. During the immersion period, a variety of data collection methods were used. These are described using Wolcott’s experiencing-enquiring-examining framework.

**Experiencing.** The researcher attended 32 practices and 16 matches involving the U13 and U16 squad over the course of the season (approximately half of the total number of training sessions and matches during this period). A mix of targeted observations—guided by the CMONs—were alternated with more casual engagements, where the researcher adopted a broader outlook. This mix of approaches helped the researcher to “make the familiar strange, and the strange familiar” [5] (p. 89).

**Enquiring.** Over the season, the researcher regularly spoke to coaches, parents and players, with conversations ranging from one-liners said in passing and casual 5–10 min courtside conversations to formal in-depth interviews. Some specific interactions were initiated by the researcher to address a specific agenda and shed light on a particular CMON. All observations and interactions were recorded as fieldwork notes and subsequently transcribed using a word processor to generate over 250 pages of double-spaced data.

**Examining.** The researcher reviewed all available documentation of the club’s history, goals, philosophy and values from the club website (i.e., club statement, club philosophy, coach, parent and player codes of conduct, club safeguarding policy, etc.). Additional club promotional materials were also collected (i.e., club leaflets, holiday camp brochures, and information circulars). All these supplementary sources of information were analysed in relation to the identified CMONs looking for corroboration or discrepancies.

### 2.5. Data Analysis

Once data were collected, the analysis took place. It progressed from the generic to the specific and back to the generic. First, the researchers interrogated the extent to which the proposed categories of mechanisms presented in part 1 were seen in the day-to-day activities of the club. A deductive-inductive analysis was conducted using NVIVO-10 [10]. Once specific mechanisms were established, the researcher attempted to link them to the outcomes—positive and negative—and contextual features described in stage one. The final analytical step integrated the findings from both stages of the project, aiming to develop a holistic, systemic understanding that supported the presentation of a theory-led, practitioner-oriented model. Within this was a commitment to identifying generic good practice, higher-level principles that might inform specific developmental practices in other sport-based environments.

### 2.6. Reflexivity

In conducting the ethnography, the issue of reflexivity was also explored and considered. Reflexivity is the process and practice of becoming aware of how researchers’ personal histories, expectations, resource opportunities and constraints impact the way they conduct and interpret research [9]. The issue of the researcher as insider/actor/agent, far from denuding the value of ethnography-based research, offers the opportunity to explore and attempt to grasp situations and occurrences which other approaches cannot begin to apprehend [5]. It is important, however, that researchers using this approach place themselves within the context of the investigation and openly acknowledge their relationship with others within it [9,11]. Although ethnography will always feature some idiosyncrasies [5], the ethnographer must present as much evidence as justifies making specific claims.

## 3. Results

Findings are presented in three sections. First, we introduce the confirmatory analysis of the generative mechanisms, contextual features and outcomes following the immersive period. Second, we describe the idiosyncratic nature of the experience and the contrasting ways in which each young person engages with the club’s developmental systems. Finally, by bringing together the findings across both phases of the study, we aim to present an evidence-based model of psychosocial development in this setting. For economy, observational notes and participant quotes have been minimised. Pseudonyms and unique identifying events are used to prevent the identification of individuals. In the case of club co-founders Coach Jack and Coach George (pseudonyms), identifying them and their real name is easy to do, given that it is feasible to determine the lead author’s club affiliation. Thus, explicit permission has been sought and granted from them to publish this paper.

### 3.1. Confirmation of Generative Mechanisms, Contextual Features and Outcomes

**Figure 1:**
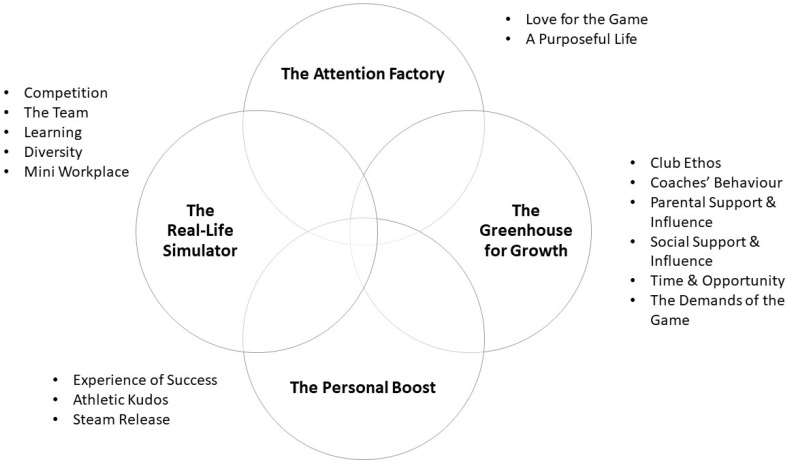
The four families of generative mechanisms of psychosocial development in sport (post Phase 1).

Part 1 identified four major families of mechanisms responsible for driving or precluding psychosocial development in sport: (i) The Greenhouse for Growth; (ii) The Personal Boost; (iii) The Attention Factory; and (iv) The Real-Life Simulator (Figure 1). ‘The Greenhouse for Growth’ revolves around the setting features that create a context in which young people secure personal growth. ‘The Personal Boost’ focuses on the distinctive capacity of participation in performance sport to generate elevated states of mind (i.e., happiness, joy, satisfaction, elation, pride, etc.). ‘The Attention Factory’ relates to the notion of the club’s ‘way’ of doing sport participation, providing athletes with a clear focus in life that activates personal agency. Finally, ‘The Real-Life Simulator’ is linked to the idea that participation in youth performance development sport intentionally and unintentionally simulates elements of adult life.

#### 3.1.1. The Greenhouse for Growth

The notion of the club as a space endowed with fertile developmental ‘soil’ and optimal ‘weather’ for growth was confirmed during the club visits. A further two mechanisms were identified, namely, time and opportunity and the demands of the game (Table 1). In this section, we review each mechanism as they were encountered in the field.

The club ethos became an inescapable theme. Underpinned by the club’s mission ‘to provide opportunities for all sections of the community regardless of ability, background, gender or age’, it could be said that the club’s modus operandi revolved around two key elements: (i) prioritising the development of the human over the player; and (ii) high, non-negotiable expectations that all stakeholders contribute to this overall outcome. Observations revealed the key role played by the club founders in maintaining a focus on this big purpose. Former high school teachers, Coach Jack and his wife Mary, and Coach George (all of them in their 70s) set the tone for the rest of the club. Jack’s attitude, especially, was repeatedly highlighted as the club’s cornerstone. As a score-keeper and guardian of the club standards, Jack was known for upholding strong values and willingly enforcing them with no concern for his public image:

“Look, he can be a pain in the backside, but someone has to be prepared to do that or the club will go down the drain. He was out for a few months because of [health issue] and you could see the club starting to slip up because no-one was prepared to do what he does. Not even Coach George (co-founder) would be able to do that.” (Coach James)

In addition, coaches’ behaviours were consistently seen as central to the greenhouse effect. Notwithstanding this, parents expressed a clear preference for the coaches they favoured for their children. Parents saw older coaches like Coach Jack, Coach George and Coach Dean as more encouraging and personable. In contrast, some of the younger coaches were deemed “too in your face and aggressive” (Sophie, parent), which risked damaging individual players’ self-esteem, which was typically regarded as fragile. Observations during matches and training sessions confirmed the existence of these two relatively different approaches to coaching. As they got older, however, players favoured the more aggressive and “army-sergeant like” (Julius, parent) approach. Michael, one of the U16 players, said:

“I love Coach Marvin’s style. He is pushing us very hard because that’s what we need now. We need to be in shape and be strong physically and mentally. If we can’t cope with training, how are we going to cope with the games?”

Notwithstanding these preferences, coaches whose style met the needs of specific players were generally described as inspirational, facilitative and as great role models. Jennifer, one of the mothers, explained:

“Coach George and Coach Dean were tremendous for my son. They turned a shy and timid boy into a happy and confident lad just by guiding him carefully and showing him a lot of love. They are wonderful role models.”

Parental support and influence were also corroborated during the immersion period. Parents were constantly seen contributing to the functioning of the environment or, as Coach Jack put it, “mucking in”. This included undertaking the duties of team manager, bus driver, fund-raiser, and many other chores. For some parents, this became almost a part-time occupation. U13 Team Manager Natalie is a prime example, as can be seen in the following:

Observer notes, 10 March—Training session 6–8 p.m. at the club:

Natalie, the team manager for the U13s, makes me giggle. Perhaps I’m being judgemental but it’s amazing what people will do for their kids. Natalie is in her mid-40 s, quite overweight and sporting a bright spray tan, full make-up, extremely long nails and massive earrings. Surely, she doesn’t belong on a basketball court, but here she is, running around the place in her heels collecting subs, sorting out transport for the weekend’s game and organising kit and refreshments. And she is loving it and thriving in this role. When I ask her about it, all she says is: “I love it. I’d do anything to help my kid, but I actually enjoy dealing with all this, I’m quite bossy you know”. Then she runs away chasing some parent who hasn’t paid yet.

The notion of social support and influence also featured strongly during live observations. The community hub nature of the club manifested itself at different levels. Club members used the facility more as a traditional youth club than as a performance sport club. Young people were seen constantly hanging around the centre, spending inordinate amounts of time “just chilling and meeting people” (Kyle, player). Thanks to its open-door policy, players came and went constantly from the moment school finished to the moment the building closed. Often, players had to be chivvied off the premises by the coaches. Likewise, the co-educational nature of the club, which did not feature in Phase 1, became prominent in Phase 2. Parents saw this positively: “look, they are 15, they are going to be into girls, I’d much rather they do it here in this safe space where we know the girl and we know their family too and what they are into” (Megan, parent).

Two new processes were, however, elicited in Phase 2. ‘Time and Opportunity’ relates to the large amounts of time players spend at the club and the opportunities this afforded for positive change. During Phase 1, time was construed as a constraint with implications for school, social and family life. During the immersion period, interpretations of time shifted to one of being much more of an enabler of opportunity; it was because of time, especially ‘better-than’ time, that participation at the club produced positive personal development. Jerome, a parent, put it like this: “any time spent at the basketball centre is better than what they could be getting up to in the street corners or on the PlayStation”.

The second novel mechanism elicited during Phase 2 related to the impact of the traditions and rules of the sport. As defaults, these acted as silent, organic mechanisms to support personal development. For instance, basketball rules place special importance on the behaviour of players and coaches, strictly enforced by referees in all games; this is accepted by all as part of the game. As one of the parents put it: “basketball people are nice; football is horrible, our son used to play football too and they get away with blue murder.” (Nadine, parent). Moreover, the game mandates other positive traditions such as the respectful ritual handshakes pre- and post-game with opposition and referees and the relationship-building hosting of a home-made finger buffet for the visiting team after each game.

#### 3.1.2. The Attention Factory

Participation in this basketball club was seen by stakeholders as providing young athletes with a clear and sustained focus. This sustained engagement was viewed as a protective shield, deterring against negative attitudes and lifestyles. In Phase 1, two major sets of sub-mechanisms were proposed: (i) love for the game and (ii) a purposeful life (Table 2). While these elements were corroborated across the immersive season, on-site observations and discussions with the stakeholders provided further insight and texture.

Player’s ‘Love for the game’ was the most discussed and observed mechanism throughout the ethnographic period. Parents regularly reported how their kids were enthralled by the sport. Jennifer, one of the mothers, shook her head while she said: “all my son wants to do in his spare time is basketball, come to the centre, in the back garden, in videogames, watching it on TV or YouTube, that’s all he has time for”. In this narrative, passion for the sport was seen as the first step in achieving a host of positive outcomes, including protection from typical dangers and distractions of the teenage years or the development of a healthy lifestyle. Nevertheless, for some parents, this passion became a liability in its own right. Sophie explained:

“His newly found ability in basketball has given him a lot of confidence and self-belief, but at the same time, that’s all he wants to do, he is not motivated to study more or put the same level of effort into anything else.”

Likewise, the notion of basketball providing ‘A purposeful life’ was salient during the immersion period. Most players expressed the ambition of securing a US college scholarship to represent Great Britain and play basketball professionally. This desire provided clear life goals, focus, purpose and direction. With such ambitions, first steps were acknowledged; do well in their GCSE exams (i.e., end of compulsory education in the UK at 16 years of age) and do extra work to refine basketball and physical skills. With this ‘purpose’, both parents and players recognised the value of the club for providing and requiring engagement in demanding, positive structures and routines. Kenny, a player for the U16 squad, clearly describe this impact:

“I know that the days I have training, I have to get well organised, come from school, get my homework done straight away, have some tea, rest, and go to training. I know that by the time I come back, I’ll be knackered and good for nothing but food and bed.”

#### 3.1.3. The Personal Boost

The capacity of sport participation to generate elevated states of mind and increased player wellbeing and intrinsic motivation was regularly confirmed during the ethnography. Phase 1 interviews established three sub-themes: (i) experience of success; (ii) athletic kudos; and (iii) steam release. The immersion period elicited a further central component: fun and enjoyment (Table 3).

The experience of success was consistently reported as imperative for securing many of the positive outcomes achieved by these young athletes; for instance, it was reported to be necessary to the development of the self (i.e., self-esteem, positive identity) and for enhanced social development (i.e., belonging). At this club, success was experienced at various levels. Of course, winning matters at this club. The club’s record of over 60 national titles in the last 20 years is unmatched nationwide. Success was also enjoyed vicariously by parents. Julius, a U16 parent, said in this respect: “every day (they) walk into the centre and look up and see the banners on the wall with all the titles. They want to have their team’s name on that banner next year, every year”. The presence of these banners and the attention paid to them provided further reinforcement about the importance of committing to working hard to play and do well.

Success was also experienced at the individual level through the constant provision of opportunities for players to improve (and to prove) their competence. Club officials continually looked to create extra opportunities for high-quality competition to stretch the players. These include opportunities to ‘play up’, playing against older and more experienced teams, organising competitive tournaments, and creating their own internal leagues when external competitions were insufficiently challenging. Over the course of a season, coaches regularly and deliberately put players in situations well beyond the players’ comfort zone; knowing that coaches only did this with adaptable players helped to create player confidence, which was then amplified by good experiences and positive reflections on the experience.

In relation to athletic kudos, players reported that playing basketball increased their ‘street cred’ (Israel, U16 player). Being recognised as a good basketball player afforded them a powerful social cache. For instance, Kenny, a bubbly and lanky 15-year old with a reputation for being difficult to manage, saw basketball as his ‘saving grace’ in school:

“I got into a bit of trouble in school, being a bit stupid you know, trying to get attention, but because I am a good ball player they let me get away with some of it, they need me for the basketball team you know.”

Players reported that being good at basketball made them unique amongst their peers. For example, when they met new people through non-basketball friends, they were introduced along the lines of “this is the basketball guy I told you about”, and this, for them, was “cool” (Israel, U16 player). Often, their superior height, which may have been a negative factor in younger years, had become a real asset in their teens. Jayden (U16) jokingly explained it as becoming “a bit of a chick-magnet”.

As for the potential for sport to act as a release valve, players were observed to arrive at the centre shortly after school finished to spend in excess of three to four hours a day on the premises. This was most common in the older groups, who were free to travel independently; they would use that time in team training, doing self-training, ‘shooting around’ or just chatting to others until 10 p.m. Training was characterised by high levels of exertion, regardless of age group. The fatigue this produced, and the need for quality sleep, helped to ensure that players would have little time and energy to dedicate to other activities (positive or negative).

Finally, the quality of time spent at the club highlighted the overall experience as ‘better than’ many alternatives, especially for experiencing ‘Fun and Enjoyment’. Players were observed during self-training sessions, normally in pairs or small groups, genuinely enjoying practicing the sport and regularly smiling and ‘laughing out loud’. This fun and enjoyment was also evident in organised sessions or competitive matches. Observations of coach-led training highlighted how coaches strived to balance the fine-tuning of technical and tactical work and drills with activities where the main goal was to offer free play so players could enjoy themselves. In this context, it was common to witness player’s exhilaration and excitement.

#### 3.1.4. The Real-Life Simulator

Many of the features of participation at the club resembled elements of the real world. ‘Adult-life-like’ situations were presented regularly and often to players at earlier ages than most of their non-club contemporaries. These experiences were laden with real meaning and consequences but were possible because of the psychological safety built through regular exposure to the club environment. The major themes proposed during phase one were confirmed in stage two and included: Competition; The Team; Learning; Diversity; and Mini-Workplace (Table 4).

Parents and players saw the club environment as “loaded with competitive pressure from multiple angles” (Amy, parent). Pressurising elements included internal competition (i) for securing selection to the weekend squad, then (ii) for playing minutes during matches, and (iii) to perform well during training and matches. Wider forms of ‘pressure’ arose from the uncertainties of the annual review to retain squad membership in the following season, competitive selection for Great Britain squads, and the challenges around securing a US scholarship. While acknowledging the inherent difficulties of dealing with this volume of pressure at early ages, both parents and players felt it fostered positive development because it was framed by the support provided by the club’s day-to-day ways of working. Many offered a relatively stoic view of this phenomenon:

“…pressure is part of life. They have to get used to it and learn to deal with it, with the failures and the disappointments. Better to do it in this safe environment than waiting to be out in the real world.” (Eva, parent)

Players were equally pragmatic and stressed that “if you can’t take the pressure you shouldn’t play national league” (Jayden).

With regard to being part of a team, the opportunity to learn to work as part of a group was widely valued. Understanding different roles, hierarchy, and the importance of discipline and doing one’s job well to maximise the chance of group success were all highlighted. Coaches consistently reinforced good team behaviours and readily reprimanded negative alternatives. For example, during one training session, a player threw a tantrum after a series of bad plays. The coach immediately stopped the session and shouted: “Who do you think you are? This is not about you, this is about the team, and what you are doing now is hurting the team!”. Being part of a team also created opportunities to lead others. This experience was highly valued by parents and players, as explained by U16 player Mikael:

“This year I have had to step up. I wasn’t the new guy anymore and there was less slack. I feel I have become much more of a leader; I have learnt to talk to people and reason with them to get them to do what they need to do for the team.”

The immersion period not only confirmed but also offered extra detail on how participation increased players’ learning capacity. A number of recurrent practices, however, did not appear to align with current learning theory; coaches often used lengthy verbal explanations, and many chose to dictate every move, allowing little room for players to contribute. New technologies (e.g., iPads, video analysis, etc.) were rarely used in teaching/coaching; instead, coaches relied on formal instruction. During ‘coaching episodes’, players were generally in one of two groups: those who seemed fully engaged, and those who appeared disengaged and even bored. Often, coaches made no clear effort to engage the disengaged players. Likewise, some players displayed sub-optimal learning attitudes, including poor listening skills and/or reluctance to accept external feedback.

Paradoxically, however, players relished every chance to direct their own learning when they were away from coaches and regular team sessions. By observation and in conversations, players were deeply committed to improving their skills. Players were seen videoing each other so they could analyse their own performance to find areas for improvement. Players watched YouTube videos of NBA player workouts to pick up drills they could try on their own. They were also regularly seen peer coaching and were comfortable with peer learning. Community leagues and holiday camps were also fertile ground for peer coaching and for players to explore their skills in a less controlled and structured setting.

Regarding diversity, Phase 2 showed the broad demographic range of individuals in the club environment. At the time of the study, club coaches came from the UK, USA, Iran, Lithuania and Spain, and the senior team included players from the UK, USA, Spain and Greece. In the national league programme, many players belonged to migrant families who came to the UK as a result of the 2008 recession (particularly from Poland, Spain and Portugal, where basketball is a mass sport). In addition, many players had roots in former British colonies, especially from the Caribbean and Africa. Moreover, diversity was enhanced by competing against, and meeting with, players from different English cities during national league games and in international tournaments. For parents and players, the developmental benefits of this situation included: (i) experiencing different cultural approaches to life; (ii) broadened horizons; and (iii) learning to deal with a variety of people and situations.

“It was just great to see him interacting with all these different people. Where we live and where he goes to school most people are white middle class and he built some great relationships with kids that he would have never met otherwise, and I think that has stood him in great stead going forward to uni and now work.” (Mark, parent)

Finally, regarding the club environment as a mini-workplace, the ethnographic period revealed that few players were taking direct advantage of these opportunities. Opportunities for work came with refereeing and coaching in the community leagues and camps, contributing to cleaning the centre, and volunteering during club events. A small group of players actively pursued these opportunities; through them, they could practice basic workplace skills and attitudes, including time-keeping, planning, responsibility and accountability.

The ethnographic period thus served to confirm, refute and refine the findings of Phase 1 of the case study. Although the mechanisms are presented as four discrete categories to ease understanding, on the ground, they represent a complex network of interdependencies and causal relationships. Mechanisms interacted in multiple ways to foster visible developmental outcomes. Two major principles appear to govern this network: (i) specific developmental outcomes are affected by multiple mechanisms; and (ii) specific mechanisms influence many networks, meaning they may simultaneously contribute to generating a number of developmental outcomes.

### 3.2. The Idiosyncratic Nature of Development

The process of apportioning causality is complex, and participant and researcher interpretation is required to connect the developmental dots. Findings from this current study confirm that personal development in this youth performance setting was significantly more nuanced and textured than previously described. The fine-grained detail provided by this case study enhances our understanding of this phenomenon. In the following section, focus is placed on the different ways in which sport was experienced and used by different athletes.

Phase 1 concluded that the experience of this programme was unique to each player. Phase 2 confirmed that the internal and external assets of the young person strongly influenced the activation of causal mechanisms and thus the generation of outcomes. Considering the overall findings, four processes were identified to explain this differential: (i) few mechanisms were universally available in equal measure; (ii) no available mechanism was optimised by all participants; (iii) psychosocial outcomes of participation were mediated and moderated by individuals’ social environments; and (iv) critical life incidents conditioned the personal narrative and the meaning of the experience. These principles will be elaborated upon separately.

#### 3.2.1. Few Mechanisms Were Universally Available in Equal Measure

Examples of mechanisms affected by this included parental support/influence, coach behaviours, social support/influence, athletic kudos, mini-workplace and diversity. We concluded that the availability and causal strength of mechanisms tended to be modulated by a series of factors. For instance, some parents were unable to regularly support their child for multiple reasons: i.e., being a single-parent family, caring for other siblings, work commitments, or compromised finances. This created impacts at different levels and had both positive and negative effects. The story of Aki illustrates this point.

Aki lives quite a few miles away from the basketball centre in a different town. His parents are immigrants from an African country, work evening jobs and do not own a car. He takes 3 separate buses (a 75-min journey in total) to get to basketball. Aki says that this has made him resourceful and self-reliant, because he cannot just sit around and wait for his mother and father to take him to baskestball. He wishes his parents came to basketball more, however, because he says they struggle to understand why he loves it so much and have tried to talk him out of it a few times; they think it may harm his education.

#### 3.2.2. No Available Mechanism Was Optimised by All Participants

Specific conditions either initiated, postponed or prevented the activation and optimisation of some generative mechanisms. These conditions typically related to the young person’s internal characteristics or assets. Playing level and progression potential are prime examples. High-ability players secured and enjoyed more playing time and, resultingly, higher status. In a virtuous spiral, these players had more exposure to success, meaning their popularity and public and self-esteem rose again. Likewise, coaches saw players’ emotional and cognitive maturity as central to activating some positive mechanisms. Players could be exposed to similar contexts, yet their individual developmental status could produce widely differing outcomes.

“They are all so different and react to things in such a different way that this helps them understand emotions much better. The kids come from very different backgrounds and have very different coping mechanisms.” (Coach Carl)

#### 3.2.3. Psychosocial Outcomes of Participation Were Mediated and Moderated by Individuals’ Social Environments

The quality and intensity of the engagement with these additional contexts plays a powerful role in determining, mitigating or enhancing the impact of sport participation. The club environment either reinforced outcomes that were primed by or already well developed by other environments. For instance, U16 parent Jamal said: “Yes, the club has taught some very good values to Simon, but they ain’t no different to the values we have tried to teach him at home. We wouldn’t have him here if that weren’t the case.” In contrast, for other players, participation in the basketball club compensated for deficiencies in other settings. Antonio, a U14 player who had recently arrived in the UK from a southern European country, was a perfect example. His mum and dad explained:

“Thank goodness for basketball. The poor child arrived with very basic English and terrified of having no friends in this cold country. He was a real sod on the plane and for the first couple of weeks of being in England, until we found this place. The moment he stepped through the doors here his face lit up. He has made lots of friends, regained a lot of confidence and learnt lots of English.”

#### 3.2.4. Critical Life Incidents Condition the Personal Narrative and the Meaning of the Experience

The second phase of the case study offered a privileged window into the personal journey of a number of players and their families who had experienced ‘critical life incidents’ (CLIs). These CLIs included loss of a sibling or parent, parental divorce, family illness, moving to a new country, bullying in school, socioeconomic deprivation, or social isolation, and each played a central role in their personal narrative and how they interpreted their sport experience. CLIs created a strong personal narrative whereby sport was framed as bringing a sense of accomplishment and justice. The narratives found at the club tended to fall into one of two categories: (i) ‘reconstruction’, which was defined as shifting from pain and suffering into happiness and accomplishment; and (ii) ‘against the odds’, which was defined as moving from humble beginnings or disadvantaged situations to high achievement. The below examples portray both narratives.

**Reconstruction:** Two families in the U13 squad, Amy and Andy and Patricia and Matthew, told harrowing stories. Amy and Andy’s family endured first the loss of a sibling. Then, Amy developed cancer, and recently, a young sibling was badly burnt in a home accident. Matthew and Patricia went through a very traumatic divorce from Matthew’s dad, a violent drug addict. In both cases, basketball became the vehicle through which they tried to rebuild their life. The three key mechanisms linked to this reconstruction process included spending as much time as possible at basketball, finding solace in the social network provided by the centre, and, especially for Andy, experiencing success and enjoyment on a regular basis.

**Against the odds:** Mikael, a U16 player that had recently migrated to the UK from another European country, provides an example of this narrative. His family originated from a war-torn African country and had fled to Europe looking for asylum. When he arrived in the UK, he spoke no English. Mikael explained how he had always felt he had to work twice as much as everyone else to show them all that he was a worthy human being. In basketball, he had found a welcoming environment where he could excel and be supported by likeminded young people that took him under his wing. Mikael threw everything into basketball, and that ‘under siege’ mentality never left him. Now 18, he is at university and working at a sports goods superstore in town. Basketball was the catalyst for him.

### 3.3. A Summary Model of Psychosocial Development in a Performance Development Club

**Figure 2:**
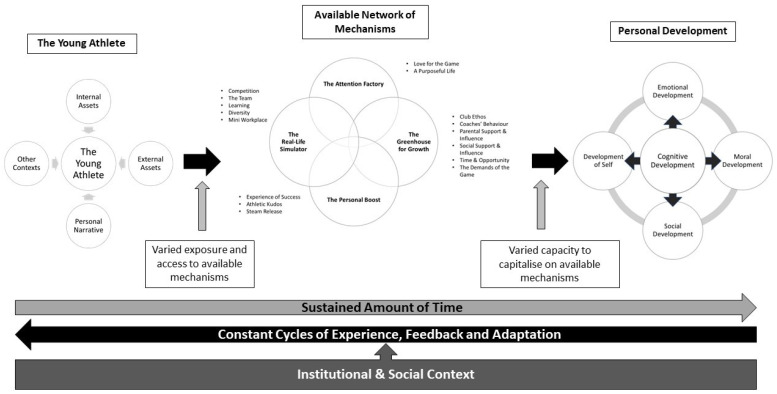
Psychosocial development model in a performance development basketball club.

Having established a wide range of outcomes, mechanisms and context networks and determined the idiosyncratic nature of development, the next section will present an integrative summary model of psychosocial development as seen in this basketball club (Figure 2). The model brings together the findings of both phases of the study in a coherent and practical way to aid sport stakeholders in making sense of the full range of factors and processes involved.

#### 3.3.1. The Young Athlete

The model starts by acknowledging that the outcome of the experience of sport is significantly influenced by the internal and external assets at the young person’s disposal, the influence of other developmental contexts, and the personal narrative attached to the sport experience. Any of these factors may be activated to direct specific generative mechanisms to bring or inhibit valued developmental outcomes. Given the range of experiences and the ways in which club experiences may cause activation, every sport experience and trajectory is highly individualised.

#### 3.3.2. A Network of Mechanisms and Outcomes

Four major families of mechanisms were elicited: (i) The Attention Factory; (ii) The Greenhouse for Growth; (iii) The Personal Boost; and (iv) The Real-Life Simulator. *The* Attention Factory comprises all mechanisms that direct and enhance the players’ focus and thus provide clear direction and objectives in sport and beyond. The Greenhouse for Growth is linked to mechanisms that provide ‘fertile ground conditions’ for positive development, especially such as the club ethos, atmosphere and social support; all require player compliance, meaning that ‘growth’ is readily and easily tested and seen. The Personal Boost refers to features of the experience that increase self-worth, self-esteem and greater personal wellbeing. Lastly, The Real-Life Simulator relates to exposure to situations that replicate adult life. This experience, supported by club-based adults, prepares players to better resolve them in the future.

Although presented in four discrete categories, this case study shows that these mechanisms form a complex network. Mechanisms interact and catalyse in multiple ways to foster or preclude developmental outcomes. Two major principles govern this network: (i) single developmental outcomes are typically affected by a combination of multiple mechanisms; conversely, (ii) single mechanisms are characteristically involved in multiple networks responsible for different outcomes. Establishing causality is challenging and very likely futile if the aim is to find ‘silver bullet’ solutions. Psychosocial development in youth performance settings is a complex multi-level and multi-directional process. The nuanced analysis provided by this case study, however, contributes to making its components more accessible and intelligible.

#### 3.3.3. Personal Development

This investigation confirmed the suitability of the psychosocial developmental outcomes framework created during the literature review stage (see part 1). The five categories of cognitive, emotional, social, moral and self-development appropriately encompass the majority of athletes’ personal outcomes. The study also confirmed a significant level of interdependence between developmental outcomes whereby some outcomes are gatekeepers to the generation of others. Notwithstanding this, each developmental outcome was linked to a set of mechanisms and the preconditions required for their activation and utilisation. The traditional realist evaluation nomenclature of context-mechanism-outcome configurations and catalogues was substituted by the newly coined term ‘context-mechanism-outcome networks’. This term brings to the fore the ambiguous reality of social life where multiple preconditions and generative mechanisms constantly interact in the production of outcomes over time.

#### 3.3.4. Time and Recurrent Experiences

This research underlines the utility of additional time (frequency and duration) spent on engaging with the sport experience. For any type of change or adaptation to occur, sustained exposure to a stimulus is required. The club environment was stimulating in many ways, especially by being time-affluent and experience-rich. Moreover, this near-limitless supply of time facilitated players’ engagement in endless cycles of experience, feedback and attitudinal and behavioural adaptation, while being diverted from other unhelpful behavioural choices. By spending more time at the club experiencing positive growth, they became progressively more skilled in capitalising on opportunities for growth. For players experiencing negative adaptations, opposite pathways prevailed.

#### 3.3.5. The Context

A final piece in the development jigsaw is the multi-layered nature and textured influence of the context for creating and moderating conditions that lead to positive personal development. Pre-existing individual characteristics determine the starting point for growth at the point of joining this club. From there, players engage with the inevitabilities of the club’s day-to-day routines, and this activates important developmental mechanisms. The number and nature of players’ interpersonal relationships within the club and beyond the club combined to impact development. Unsurprisingly, coaches, parents and peers played an essential part in socially-based development. In addition, specific institutional characteristics, including the non-negotiables of a strongly humanistic club philosophy, its ‘cross-roads’ geographic location, and a longstanding commitment to competitiveness, all contributed systems that mandated responsiveness and, therefore, personal development. Likewise, the infrastructure, understood as the external conditions—including levels of funding—all impacted the way the sport was, and this coloured the way players developed. Specifically, the minority and non-professional character of basketball in the United Kingdom created additional drivers for self-sufficiency, resilience and proactivity, all important markers of personal development.

## 4. Discussion

### 4.1. The Nature of Development

The findings of this case study show psychosocial development as a conditional, multi-faceted, time-sensitive and highly individualised phenomenon. Given the relevance and the essential dynamism of context, offering a deterministic and infallible view of the development process is futile. Positive development can thus be considered a ‘wicked problem’ (i.e., a problem with multiple interdependencies, no single solution, and no clear stopping point) within a complex system [12]. Nevertheless, this study has shown the development process can still be reduced to a set of basic principles that can be transferred to diverse settings and contexts. A wide-lens approach has been adopted to distil these key principles and processes to generate a synoptic view of psychosocial development in sport. This evidence-based catalogue of options can inform sport psychologists, coaches and all other stakeholders in the creation of psychosocially ‘competent environments’ [13] to support development in young people.

The study shows the cyclical, repetitive and sustained nature of the development process—a system of repetition. It suggests a constant flow of interactions between the individual and the environment and acknowledges the mutable condition of the individual and the setting. Heraclitus’ famous aphorism, “No man ever steps in the same river twice, for it is not the same river and he is not the same man” [14] (p. 29), could be adapted for youth sport to read “No young person goes to the same club twice, for it is not the same club, and they are not the same person.” Psychosocial development is thus the result of ongoing and multi-level cycles of experience, feedback, reflection and adaptation players consciously and subconsciously engage with during their participation at the club which, over time, and in line with previous studies, lead to significant and long-lasting changes [15,16,17,18,19]. Notably, we show this affecting not only players but also parents and coaches.

### 4.2. The Process of Development

The present study contributes to advancing the field and supporting practitioners on the ground by identifying four processes (or families of mechanisms) that mediate development.

#### 4.2.1. The Attention Factory (Attentional Focus)

The study suggests that the compelling effect of the sport—its power to grab the young players’ attention and imagination—was central to its success. Without focused attention and commitment to the activity—deep attention—the impact is reduced due to lack of meaning and significance [20,21]. Innovatively, the study identified that attention is driven by two parallel processes: first, a basic stimulus-reward system, where certain ‘reward-rich’ activities and features ‘hook’ athletes to the setting; and second, the life-affirming nature of other mechanisms, which provided a clear and distinct life purpose. These were magnified by the socially satisfying elements of that experience (i.e., belonging to a cohesive group). Attention can thus be driven both autonomously, quasi-organically and deliberately through the systems put in place by key stakeholders.

#### 4.2.2. The Real-Life Simulator (Structured and Unstructured Skill-Building Activities)

Once attentional focus is achieved, skill-building activities are essential to ensure development [22]. Some of these activities can be structured and planned to deliver clear personal development goals (i.e., classroom-based workshops, doing chores that contribute to the running of the club, etc.). Other activities may be more organic and unstructured in nature (i.e., inherent diversity at the club, exposure to teaching, the demands of the game, etc.). Significantly, the present study shows that repeated and consistent exposure to relatively low-key activities within this club was important and successful for building individuals’ internal assets. This underlines the ongoing debate about the extent of development arising from serendipity versus ‘by design’ activities [23,24,25,26,27]. The evidence developed through this study suggests that both pathways coexist and should be addressed, if only because the pathways to impact are so complicated and hard to isolate [23].

#### 4.2.3. The Greenhouse for Growth (Deliberate and Incidental Support)

Having a supply of personal and personalised support appears to modulate psychosocial development in sport [28]. An explicit humanistic philosophy embodied by all stakeholders, ensured by recruiting individuals with a genuine caring disposition into key positions, especially coaches and club administrators, guarantees that few players could consistently escape, or purposely avoid, this experience. Support may be provided in two ways. It can be deliberate and purposeful, built into the routines of the adults in the setting (i.e., welcome meetings, discussion sessions, general and casual check-ins, crisis management help, and regular communication with families). Other approaches may occur within the spontaneous functioning of the networks of club supporters, including parents (one’s own and others’) and all other social actors within the setting, including teammates and club members. Together, all these elements enhance personal wellbeing, encourage players to be ever-ready to capitalise on any development opportunity, and buffer against the negative effects of participation [23,29].

#### 4.2.4. The Personal Boost (Feelings of Personal Growth)

The fourth developmental process involves regularly experiencing feelings of personal growth. A consistent sense of betterment and achievement has been shown to encourage recommitment to the activity, creating a virtuous cycle of engagement and re-engagement [30,31]. Over time, recurrent and multiple opportunities to interact with the various developmental mechanisms available in the club increase the chances of practicing different developmental behaviours [29]. These feelings of growth come from a variety of sources. Players were provided with regular opportunities to experience tangible success, personal kudos, and the increased sense of personal worth and identity gained from belonging to a large community of interest.

Taken together, the process of personal development can be summarised using the unattributed adage, “The best gift we can give our children is roots to grow and wings to fly”. Basketball provided the attentional focus, and the deliberate and incidental support provided the roots. The ground conditions provide motivational nutrients and a stable climate to support growth. Complementing these conditions, both structured and unstructured skill-building opportunities and the regular provision of feelings of personal growth equipped players with the confidence to grow their wings and fly towards better versions of themselves.

### 4.3. Differentiated Impact—The Young Person Who Was, Who Is, and Who Will Be

Despite evidence of the co-existence of the above-described processes, the impact of the experience is widely divergent. In line with recent studies, this paper refutes the naïve belief of sport offering a ‘magic bullet’ with inherent properties that bring universal benefits regardless of how it is delivered and experienced [32,33,34,35]. Operating with distinctive thresholds and activation points, generative mechanisms were highly unique; often, it was not possible to predict their activation and, as a result, even less possible to know how players might react when they were activated [36]. In line with Bronfenbrenner’s bioecological model of human development [37], the elements young people derived from participation reflected person-specific drives and experiences. In the current study, prominent person-specific factors were internal disposition, external assets and personal narratives. Further, these factors interacted to mould the young athletes’ behaviours and interactions, depending on the settings and dynamic qualities of the context.

General and sport-specific psychosocial development literature has espoused Bronfenbrenner’s views on human development [38,39,40]. However, research in sport has tended to emphasise environmental factors [41,42,43] and the actions of significant others like coaches, parents and peers [44,45]. Few studies have addressed how any players’ initial personal characteristics influence subsequent developmental trajectories. Recent exceptions include Pierce et al.’s model of transfer in life skill development [18,36], and Holt et al.’s grounded theory model [39].

In these models, the starting point for understanding the transfer of life skills from sport to life is the internal and external assets of the young person, alongside their autobiographical experiences. The findings of the current case study align with these models. The current study also signals the need for coaches, administrators and parents to respond to individual characteristics and to create opportunities that: (i) increase the development of each player’s internal disposition; (ii) buffer any potential negative effect of external assets; and (iii) allow players to become familiar with their own personal narratives and identities. Deliberately and momentarily shifting to consider the young person who ‘was’, how they appear ‘now’ and the young person who ‘will be’, is central to supporting the dynamic, idiosyncratic processes of development.

## 5. Conclusions

This case study shows that participation in performance sport affects young people’s development. This occurs whether or not sport psychologists, coaches, programme designers, parents or players intentionally plan for it. The current findings, however, provide a comprehensive yet distinctively individualised and multi-layered picture of the conditions and processes that support positive development. They do not describe any young person’s development. The anonymous adage, “The best gift we can give our children is roots to grow and wings to fly,” summarises our findings. The generic principles distilled from this specific case provide a useful starting point for developing more programmes to support the positive development of more young players. In the wake of COVID-19, the findings may be especially relevant for supporting continued investment in and re-engagement with sport.

The study also showed the value of using a RE approach for the investigation of psychosocial development in sport. It helped to generate a detailed account of the process and also helped in understanding its idiosyncratic nature. This notwithstanding, in recognising its limitations, we can recommend future research directions. Longitudinal tracking and measuring specific developmental outcomes may yield new insights. Likewise, our limited focus on a single club, age group and gender calls for further studies. Variously, these may corroborate, refine or refute the programme theories presented in this paper. Similarly, studies focusing on the internal and external assets and personal narratives of individuals may provide additional awareness of how these elements condition the short- and long-term impact of the experience.

## Figures and Tables

**Table 1:** The Greenhouse for Growth—complete mechanisms post-immersion.

The Greenhouse for Growth
Club Ethos
Coaches’ Behaviours
Parental Support/Influence
Social Support/Influence
Time and Opportunity (new)
The Demands of the Game (new)

**Table 2:** The Attention Factory—complete mechanisms post-immersion.

The Attention Factory
Love for the Game
A Purposeful Life

**Table 3:** The Personal Boost—complete mechanisms post-immersion.

The Personal Boost
Experience of Success
Athletic Kudos
Steam Release
Fun and Enjoyment (new)

**Table 4:** The Real-Life Simulator—complete mechanisms post-immersion.

The Real-Life Simulator
Competition
The Team
Learning
Diversity
Mini-Workplace
